# Prevalence and Predictors of Vitamin D Deficiency and Insufficiency among Pregnant Rural Women in Bangladesh

**DOI:** 10.3390/nu13020449

**Published:** 2021-01-29

**Authors:** Faruk Ahmed, Hossein Khosravi-Boroujeni, Moududur Rahman Khan, Anjan Kumar Roy, Rubhana Raqib

**Affiliations:** 1 Public Health, School of Medicine, Griffith University, Gold Coast Campus, Gold Coast, QLD 4220, Australia; khosravi_bh@yahoo.com; 2 Institute of Nutrition and Food Science, University of Dhaka, Dhaka 1000, Bangladesh; khan.moudud@gmail.com; 3 International Centre for Diarrhoeal Disease Research, Mohakhali, Dhaka 1212, Bangladesh; anjan@icddrb.org (A.K.R.); rubhana@icddrb.org (R.R.)

**Keywords:** vitamin D deficiency, vitamin D insufficiency, hypovitaminosis D, pregnant women, Bangladesh

## Abstract

Although adequate vitamin D status during pregnancy is essential for maternal health and to prevent adverse pregnancy outcomes, limited data exist on vitamin D status and associated risk factors in pregnant rural Bangladeshi women. This study determined the prevalence of vitamin D deficiency and insufficiency, and identified associated risk factors, among these women. A total of 515 pregnant women from rural Bangladesh, gestational age ≤ 20 weeks, participated in this cross-sectional study. A separate logistic regression analysis was applied to determine the risk factors of vitamin D deficiency and insufficiency. Overall, 17.3% of the pregnant women had vitamin D deficiency [serum 25(OH)D concentration <30.0 nmol/L], and 47.2% had vitamin D insufficiency [serum 25(OH)D concentration between 30–<50 nmol/L]. The risk of vitamin D insufficiency was significantly higher among nulliparous pregnant women (OR: 2.72; 95% CI: 1.75–4.23), those in their first trimester (OR: 2.68; 95% CI: 1.39–5.19), anaemic women (OR: 1.53; 95% CI: 0.99–2.35; *p* = 0.056) and women whose husbands are farmers (OR: 2.06; 95% CI: 1.22–3.50). The risk of vitamin deficiency was significantly higher among younger pregnant women (<25 years; OR: 2.12; 95% CI: 1.06–4.21), nulliparous women (OR: 2.65; 95% CI: 1.34–5.25), women in their first trimester (OR: 2.55; 95% CI: 1.12–5.79) and those with sub-optimal vitamin A status (OR: 2.30; 95% CI: 1.28–4.11). In conclusion, hypovitaminosis D is highly prevalent among pregnant rural Bangladeshi women. Parity and gestational age are the common risk factors of vitamin D deficiency and insufficiency. A husband’s occupation and anaemia status might be important predictors of vitamin D insufficiency, while younger age and sub-optimal vitamin A status are risk factors for vitamin D deficiency in this population.

## 1. Introduction

Vitamin D plays important role in bone mineralisation through the maintenance of calcium and phosphorus homeostasis [1]. In addition, accumulated evidence suggests that vitamin D influences several pathophysiological processes and is also known to modulate both innate and adaptive immunity [2]. Vitamin D deficiency is also associated with an increased risk of several chronic diseases, such as diabetes mellitus and cardiovascular diseases [3]. Furthermore, hypovitaminosis D is associated with impaired muscle function [4,5], which (when combined with being overweight) may lead to sarcopenia [6]. While there are limited nationally representative data available, hypovitaminosis D appears to be widespread among all population groups across the globe and is currently recognised as an emerging public health problem [7].

Hypovitaminosis D is highly prevalent among pregnant women [8,9]. Vitamin D has been linked with several important functions in pregnancy, including glucose homeostasis, placental function, inflammatory response and infection control [10]. Furthermore, studies have shown that vitamin D deficiency increases the risk of adverse pregnancy outcomes, such as preeclampsia, gestational diabetes mellitus and small-for-gestational-age [11,12]. A recent systematic review and meta-analysis of vitamin D supplementation during pregnancy demonstrated a significant increase in mean birth weight, reduction in the risk of small-for-gestational-age, increase in length at one year of age and reduction in the risk of offspring asthma or recurrent/persistent wheeze up to three years of age [13].

In Bangladesh, previous studies have shown a high prevalence of vitamin D deficiency among non-pregnant women [14,15,16,17] and postmenopausal women [18]. Three studies have reported the prevalence of vitamin D deficiency among pregnant women living in Dhaka city, Bangladesh [19,20,21]. Although there are limited data on vitamin D status among pregnant rural women, one intervention trial, with a different objective, reported vitamin D deficiency among pregnant rural women in Northern Bangladesh [22].

Vitamin D status in a population is known to be affected by various environmental and personal factors, such as dark skin, high latitude or low sunshine climates, use of sunscreen, ethnic background, certain clothing and cultural practices [3,8,14,23,24,25,26,27]. Additionally, a recent systematic review examining the effect of iron on vitamin D metabolism indicated a positive association between iron status and vitamin D status [28]. The importance of interaction between vitamin D status and other micronutrients, particularly during pregnancy, has been recognised [29]. However, to date, there is a lack of data examining the association of vitamin D and other micronutrients.

Furthermore, earlier studies have shown that serum vitamin D concentrations are influenced by the presence of inflammation or infection [30,31]. Thus, it is necessary to consider the effect of sub-clinical infection or inflammation on serum vitamin D concentration for accurate assessment of vitamin D status in populations living in low- and middle-income countries where the risk of chronic inflammation is high [29]. None of the previous studies in the Bangladeshi population has considered the effect of sub-clinical infection or inflammation while assessing vitamin D status.

This study was designed to examine the prevalence of vitamin D deficiency and insufficiency in pregnant rural women in Bangladesh, and to examine the association of various socio-economic, pregnancy and diet-related factors, selected micronutrient status and the presence of infection/inflammation with vitamin D insufficiency and deficiency separately in this population.

## 2. Materials and Methods

### 2.1. Study Participants

This cross-sectional study was conducted in April and May 2015 among pregnant women, gestational age ≤ 20 weeks, living in rural Bangladesh. Pregnant women who had already visited an antenatal clinic (ANC) for a check-up during their current pregnancy were excluded from the study to ensure they were not receiving any kind of routine vitamin and mineral supplementation.

### 2.2. Selection of Participants

Four Upazilas (sub-districts, Sharishabari, Pirgachha, Lalmohon and Badarganj) from three geographical regions (Northern, Southcentral and North-East) of Bangladesh were selected purposively. Of the four Upazilas, two (subdistricts, Sharishabari and Pirgachha) from predominantly high-groundwater-iron areas and two (Lalmohon, Badarganj) from areas of predominantly low groundwater iron, were selected. A total of 24 unions (administrative units, consisting of a cluster of villages), 6 unions from each Upazila, were randomly selected. An approximately equal number of pregnant women were then recruited from each union using a convenience sampling method. The study protocol was approved by the Ethics Committee of the Faculty of Biological Sciences, University of Dhaka, Dhaka, Bangladesh (on 16 April 2015; Ref No. *Biol. Sci.* 2014–2015).

### 2.3. Data Collection

Eligible pregnant women were identified based on the date of their last menstrual period (LMP) and the ANC visit during the current pregnancy by the field staff visiting each household. The purpose of the study was explained to all eligible pregnant women, and they were invited to attend the local ANC on a pre-set date for data and blood collection. A total of 530 pregnant women participated in the study. The overall response rate was over 90%. On the day of data collection, after providing informed consent with a signature or thumb impression, all women were tested for pregnancy at the ANC using a commercial pregnancy detection kit. The interviewer again confirmed the time of LMP. Socio-demographic (age, participants’ and their husbands’ education and occupation, household size, possession of cultivable land) and pregnancy-related information (parity and gestational age) were collected by trained interviewers. Information on participants’ usual dietary patterns was obtained by interview using a 7-day food frequency questionnaire on selected food items rich in micronutrients (red meat [beef, goat and liver], fish [small and big], dairy [milk and milk products] and eggs, leafy green vegetables, non-leafy vegetables and seasonal fruits). Data on the previous 30 days’ consumption of any vitamin and mineral supplements were also collected.

### 2.4. Blood Specimen Collection

Using a disposable syringe, a phlebotomist collected five millilitres of venous blood from each woman. Serum was separated by centrifugation and transported in plastic microcentrifuge tubes, frozen in dry ice, to a laboratory in Dhaka, and stored at −20 °C until analysed.

### 2.5. Analytical Procedures

Serum vitamin D levels were estimated by measuring total 25-hydroxyvitamin D [25(OH)D; the sum of 25(OH)D_2_ and 25(OH)D_3_] using electrochemiluminescence immunoassay on automated analysers (Cobas e601, Roche immunoassay analysers, Mannheim, Germany). This method was standardised against LC-MS/MS, which had been standardised to the National Institute of Standards and Technology (NIST) standard. According to the manufacturer, this assay showed 98% and 81% cross-reactivity with 25-OH vitamin D_3_ and 25-OH vitamin D_2_, respectively. Preci Control Varia (cat. No. 05618860 190) based on human serum was used for vitamin D as a control. One aliquot was used in each run in a day as a QC. The inter-assay CV for serum vitamin D was 7.8% (using Preci Control Varia level-1) and 2.7% (using Preci Control Varia level-2).

Haemoglobin concentration was measured by HemoCue Hb 301 hemoglobinometer (Hemocue, Ängelholm, Sweden). Serum ferritin, a marker of iron status, was measured by enzyme-linked immunosorbent assay, using commercial kits (BioCheck Inc., Foster City, CA, USA). Serum retinol (vitamin A) concentration was measured using high-performance liquid chromatography [32]. Serum C-reactive protein (CRP), a marker for acute inflammation, was measured by enzyme-linked immunosorbent assay using commercial kits (BioCheck Inc.). Serum α1-acid glycoprotein (AGP), a marker for chronic inflammation, was measured by the immunoturbidimetric method using commercial kits on a Cobas C311 (Roche Diagnostics, Mannheim, Germany) analyser.

### 2.6. Cut-Off Used to Define Vitamin D and Other Micronutrient Deficiency

There is a lack of consensus about the cut-off for defining adequate vitamin D status, specifically for pregnant and lactating women [33]. As per current guidelines, in this study, vitamin D deficiency was defined as serum 25(OH)D < 30 nmol/L, with serum 25 (OH)D levels of 30–<50 nmol/L defined as vitamin D insufficiency and serum 25(OH)D level ≥ 50 nmol/L considered as normal [34]. Anaemia was defined as haemoglobin concentration <11.0 g/dL [35]. As serum ferritin is an acute-phase protein, its concentrations can be influenced by the presence of infection or inflammation [36]. Elevated serum CRP (>10 mg/L) and AGP (>1.0 g/L) were considered to indicate the presence of infection/inflammation [34]. Thus, serum ferritin concentrations were adjusted for elevated CRP (>10 mg/L) and AGP (>1.0 g/L) concentrations by mathematical correction [37], and iron deficiency was defined as adjusted serum ferritin concentration < 15.0 μg/L [35]. Serum retinol is also affected by the sub-clinical infection, but concentration decreases in its presence [38]. Hence, serum retinol concentrations were also adjusted for CRP and AGP concentrations by mathematical correction [39]. Vitamin A deficiency and marginal/sub-optimal vitamin A status were defined by adjusted serum retinol concentration < 20.0 µg/dL and 20.0–<30.0 µg/dL, respectively [40].

### 2.7. Statistical Analysis

Statistical analyses were carried out using SPSS (version 26; SPSS Inc., Chicago, IL, USA). Fifteen subjects were excluded because of either incomplete data or insufficient blood samples for vitamin D assay. Therefore, 515 participants were included in the analyses. Normal distributions of serum vitamin D, haemoglobin and serum vitamin A concentrations were confirmed by Kolmogorov-Smirnov goodness-of-fit test. Because serum ferritin concentrations were not normally distributed, data were log-transformed. The outputs back-transformed to the original scale are presented. The univariate analysis consisted of a simple frequency distribution of selected variables.

To examine the relationship of vitamin D status with various socio-demographic factors, pregnancy, diet-related characteristics, infection/inflammation and selected micronutrient status, each variable was grouped based on a priori logical categories. An independent *t*-test or one-way analysis of variance was applied to compare the mean serum vitamin D concentrations between groups for selected variables (age, parity, gestational age, inflammation and various micronutrients’ status) as appropriate. The differences in the prevalence of vitamin D deficiency and insufficiency between various socio-demographic, pregnancy and diet-related groups, inflammation status and selected micronutrients’ status were compared by Chi-square test.

Finally, a backward stepwise binary logistic regression analysis was performed to determine the independent association of various socio-demographic, pregnancy- and diet-related variables, selected micronutrients’ status and presence of inflammation/infection with vitamin D deficiency and insufficiency separately. The independent variables included in the analysis were age, parity, gestational age, inflammation status judged by elevated CRP and AGP, anaemia, iron and vitamin A status, participants’ and their husbands’ education and occupation, household size, cultivable land ownership, intake of vitamin and mineral supplements and frequency of consumption of large fish, eggs and dairy products. For age grouping, adolescent women were combined with younger adult women as there was no significant difference in serum vitamin D levels between the two groups. Furthermore, for husband’s occupation, business and service groups were combined. The vitamin A deficiency group was combined with the marginal vitamin status group, because only a small proportion of the women had vitamin A deficiency, to increase the precision of the analysis. Results of the regression analyses are presented as odds ratios (OR) and 95% confident interval (CI). A *p* value < 0.05 was considered statistically significant.

## 3. Results

The mean (±SD) age of the pregnant women was 23.6 (±4. 8) years, ranging from 13 to 38 years. The mean (±SD) gestational age of the pregnant women was 15.17 (±2.75) weeks, ranging from 7 to 20 weeks of gestation (data not shown). Twenty-four per cent of the pregnant women were adolescents (aged 19 years or younger), 31.7% were younger adults (aged between 20–24 years), and the rest (44.3%) were 25 years or older (Table 1). More than a third (37.1%) of the total pregnant women were nulliparous. Nearly 14% of the women were in their first trimester of pregnancy (gestational age < 13 weeks), and the rest were in their second trimester. Overall, 44.5% of the pregnant women and 58% of their husbands were functionally illiterate (had never been to school or had completed up to Class V only). Nearly two-thirds (65%) of the participants’ husbands were either day labourers or farmers, while 96.3% of the pregnant women were housewives. More than half (56.5%) of the participants had no cultivable land (Table 1).

Only about 8% of the pregnant women reported consuming vitamin and mineral tablets during the previous month (Table 1). Only 7.2% of the pregnant women had acute inflammations/infections judged by serum CRP > 10.0 mg/L and only 1.6% of the women had chronic inflammations/infections judged by serum AGP > 1.0 g/L. Thirty-four per cent of the pregnant women had anaemia, 26.4% had iron deficiency, 3.1% had vitamin A deficiency and another 30.7% had marginal/sub-optimal vitamin A status.

**Figure 1:**
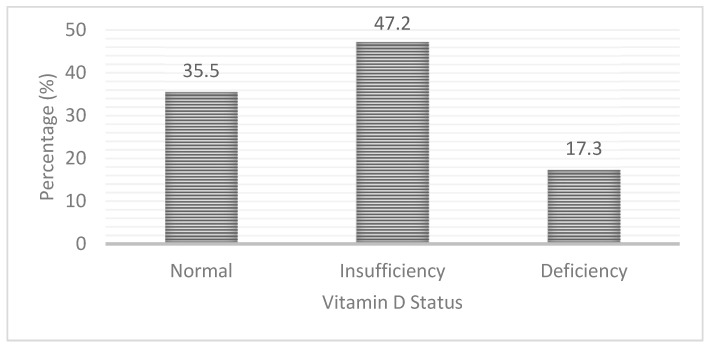
Prevalence of vitamin D insufficiency and deficiency among pregnant rural women in Bangladesh.

Nearly two-thirds (64.5%) of the pregnant women had hypovitaminosis D [serum 25(OH)D < 50.0 nmol/L] with 17.3% having vitamin D deficiency [serum 25 (OH)D < 30 nmol/L] and 47.2% having vitamin D insufficiency [serum 25 (OH)D 30–<50 nmol/L]. The rest of the pregnant women (35.5%) had adequate vitamin D status (Figure 1).

When looking at dietary patterns, more than half (55%) of the pregnant women had not consumed meat (beef and mutton) and liver, and a quarter of the women had not consumed eggs in the week preceding the interview. Close to 40% of the pregnant women had not had big fish, and/or milk and milk products in the week preceding the interview (data not shown).

Compared to the older (≥25 years or more) pregnant women, the mean serum vitamin D level was significantly lower among the adolescent (age < 20 years) and younger adult (20–24 years) pregnant women (*p* = 0.001). However, there was no significant difference in serum vitamin D concentrations between the adolescent and younger adult pregnant women (Table 2). The mean serum vitamin D level was significantly (*p* = 0.001) lower among nulliparous pregnant women than multiparous pregnant women (Table 2). The pregnant women in their first trimester had a significantly lower mean serum vitamin D level than the pregnant women in their second trimester (*p* = 0.005; Table 2). There were no significant differences in serum vitamin D concentrations between pregnant women with and without inflammation/infection. The pregnant women with sub-optimal vitamin A status had significantly lower serum vitamin D concentrations than the pregnant women who had adequate vitamin A status (*p* < 0.001). No significant differences were observed between pregnant women with and without anaemia and /or iron deficiency (Table 2).

There were significant differences in the prevalence of vitamin D deficiency and insufficiency between various age (*p* < 0.001), parity (*p* < 0.001) and gestational age (*p* < 0.026) groups (Table 3). None of the socio-economic factors was associated with the prevalence of vitamin D deficiency or insufficiency. No significant differences were observed in the prevalence of vitamin D deficiency or insufficiency between the frequency of consumption for eggs, dairy products, meat or big fish groups. Similarly, there were no significant differences in vitamin D levels between pregnant women who did and did not take vitamin and mineral supplements for one month preceding the interview (Table 3).

Table 4 depicts the differences in the prevalence of vitamin D insufficiency and deficiency among pregnant women by selected micronutrients and infection/inflammation status. No significant differences in the prevalence of vitamin D insufficiency and deficiency were observed between pregnant women with and without inflammation/infection. There were significant differences in the prevalence of vitamin D deficiency and insufficiency between pregnant women with sub-optimal vitamin A status and pregnant women with normal vitamin A status (*p* = 0.009). No significant differences in the prevalence of vitamin D insufficiency and deficiency were found between the pregnant women with and without anaemia and/or iron deficiency.

Factors associated with vitamin D insufficiency in pregnant women were examined using logistic regression analysis (Table 5). The nulliparous pregnant women were 2.72 times more at risk of vitamin D insufficiency than the multiparous pregnant women (OR: 2.72; 95% CI: 1.75–4.23; *p* = 0.0001). The risk of vitamin D insufficiency was significantly higher among the pregnant women in the first trimester than the pregnant women in the second trimester (OR: 2.68, 95% CI: 1.39–5.19; *p* = 0.003). The risk of vitamin D insufficiency was significantly higher among the pregnant women whose husbands were farmers (OR: 2.06; 95% CI: 1.22–3.50; *p* = 0.007) than the pregnant women whose husbands were in business or service. Anaemic pregnant women were at higher risk of vitamin D insufficiency (OR 1.53. 95% CI: 0.99–2.35) compared to non-anaemic pregnant women. The finding was not statistically significant; however, it approached the borderline of significance (*p* = 0.056). No association was observed between the sub-optimal vitamin A status and/or consumption of various vitamin D rich foods and vitamin D insufficiency.

A similar analysis was carried out to identify the factors associated with vitamin D deficiency, as was done for vitamin D insufficiency in rural pregnant women (Table 5). The results showed that the risk of vitamin D deficiency was 2.12 times higher among adolescent and younger pregnant women (age < 25 years) compared to older (age ≥ 25 years) pregnant women (OR: 2.12, 95% CI: 1.06–4.21; *p* = 0.033). The nulliparous pregnant women were 2.65 times more at risk of vitamin D deficiency than the multiparous pregnant women (OR: 2.65, 95% CI: 1.34–5.25; *p* = 0.005). Moreover, the risk of vitamin D deficiency was significantly higher among the pregnant women in the first trimester than the pregnant women in the second trimester (OR: 2.55, 95% CI: 1.12–5.79; *p* = 0.025). The pregnant women who had sub-optimal vitamin A status were 2.30 times more at risk of vitamin D deficiency than those who had adequate vitamin A status (OR: 2.30; 95% CI: 1.28–4.11; *p* = 0.005). None of the socio-demographic and diet-related factors was found to be associated with vitamin D deficiency.

## 4. Discussion

In this study, nearly two-thirds of the pregnant women were found to have hypovitaminosis D. Largely, these women came from lower-educated families, and were mostly housewives living in rural settings with relatively lower socio-economic backgrounds. In the present study, 37 (7.2%) pregnant women had acute inflammation/infection judged by serum CRP concentration > 10.0 mg/L, and 8 (1.6%) pregnant women had chronic inflammation/infection judged by serum AGP concentration > 1.0 g/L. Previous studies have indicated that serum vitamin D concentrations are influenced by inflammation/infection [30,31,41]. Thus, we examined whether the presence of inflammation/infection affected the serum vitamin D concentration while assessing the prevalence of vitamin D insufficiency and deficiency. Our findings revealed no significant difference either in mean serum vitamin D concentrations or in the prevalence of vitamin D insufficiency and deficiency between pregnant women with and without inflammation/infection, indicating that the presence of inflammation/infection did not affect the vitamin D status in this study’s samples. Therefore, we have reported the prevalence of vitamin D insufficiency and deficiency among pregnant women irrespective of their inflammation/infection status.

Of the total, 17.3% of the pregnant women had vitamin D deficiency (serum 25(OH)D < 30.0 nmol/L) and another 47.2% had vitamin D insufficiency (serum 25(OH)D 30–<50.0 nmol/L). An earlier study among rural pregnant women in Northern Bangladesh reported a 64% prevalence of vitamin D deficiency using a cut-off of plasma 25(OH)D < 50.0 nmol/L [22]. Another small-scale study (*n* = 140) of pregnant women conducted in Dhaka city also reported that 63% had serum 25(OH)D < 50.0 nmol/L [19]. Although we defined vitamin D insufficiency and deficiency using different cut-off values for serum 25(OH)D, the proportion of women with serum vitamin D concentration below <50.0 nmol/L is very similar to that reported in both urban [19] and rural pregnant women [22]. Conversely, a study conducted among women in their early pregnancy (6–14 weeks of gestation) in Dhaka city reported 46.4% prevalence of vitamin D deficiency using a cut-off of serum vitamin D < 30 nmol/L [21], which is more than double the prevalence of vitamin D deficiency observed in our rural study population. Nevertheless, all the above findings indicate a very high prevalence of hypovitaminosis D (serum 25(OH)D concentration < 50.0 nmol/L), irrespective of the level of urbanisation, in Bangladeshi pregnant women. Studies from India [42] and China [43] also reported a very high prevalence of vitamin D deficiency and insufficiency among pregnant women.

In the present study, we conducted a logistic regression analysis to identify the factors that are independently associated with vitamin D deficiency and insufficiency separately in this population. The findings revealed that after adjusting for the effect of various confounders, both vitamin D deficiency and insufficiency were significantly associated with parity. That is, nulliparous pregnant women were at higher risk of vitamin D deficiency (adjusted OR: 2.65; 95% CI: 1.34–5.25) and insufficiency (adjusted OR: 2.72; 95% CI: 1.75–4.23) than multiparous pregnant women. The results are consistent with earlier studies that showed primigravid women were more likely to have vitamin D deficiency than multiparous women [44,45]. In contrast, a study among Caucasian women in Belfast could not find any association between parity and hypovitaminosis D [46]. Furthermore, a study among Danish women reported a positive association between parity and risk of vitamin D deficiency, but no association of parity with vitamin D insufficiency [47]. A possible reason for the disparity in the findings between studies could be the difference in the skin colour of the studied population. Women with dark skin are known to have a higher prevalence of vitamin D deficiency compared with Western women [24,44].

Furthermore, the risk of both vitamin D deficiency and insufficiency were higher in the women who were in the first trimester of pregnancy than the women who were in the second trimester of pregnancy. These findings were similar to earlier studies [48,49]. The reason for the improved vitamin D status in the second trimester of pregnancy is most likely due to the hormonal and metabolic changes that occur during pregnancy. While the effect of pregnancy on 25(OH)D status is less well understood [50], it is thought that the presence of prolactin and placental lactogen might play roles in increasing the synthesis of serum 25(OH)D with the progress of the pregnancy, as both hormones increase throughout pregnancy, but return to pre-pregnancy values after delivery [51].

Our study also revealed that the younger pregnant women (age < 25 years) were 2.15 times more likely to have vitamin D deficiency than the older pregnant women (age 25 years or over). On the other hand, such an association between the pregnant women’s age and vitamin D insufficiency was not found. In line with our findings, a study conducted among pregnant women in a multi-ethnic community in Australia also reported younger maternal age is a risk factor for vitamin D deficiency [52]. Another study carried out with Arab women in Qatar similarly reported a higher risk of vitamin D deficiency among younger women aged < 30 years compared to older women [11]. Thus, our finding is consistent with the current literature that younger pregnant women are more susceptible to developing vitamin D deficiency. There may be several reasons for this difference, including a difference in lifestyle. Unfortunately, we do not have the data to explore this further. However, it may also be possible that the younger pregnant women may need more vitamin D to support their development (as some may still be growing), as well as for foetal growth, compared to the older women.

In the present study, we found that the anaemic pregnant women were at higher risk of vitamin D insufficiency [OR: 1.53 (95% CI: 0.99–2.35)] than the pregnant women with normal haemoglobin status. However, the finding did not reach a traditional, statistically significant level (*p* = 0.056). Nevertheless, the present finding indicates a trend for an independent association between anaemia status and vitamin D insufficiency, but no such association was observed for vitamin D deficiency. In contrast, a study among Korean adults reported a significantly higher prevalence of vitamin D deficiency in the anaemic group compared with the non-anaemic group with an odds ratio of 3.316 (95% CI: 2.265–4.854), but no significant association between anaemia and vitamin D insufficiency [41]. The discrepancy in the findings could be due to the difference in the severity of anaemia status in the studied populations. None of the pregnant women in our study had severe anaemia, while the Korean study consisted of participants with severe anaemia. In another Korean study, the likelihood of vitamin D deficiency (serum 25(OH)D < 50 mmol/L) was 4.12 times higher (95% CI: 1.665–10.171) in infants with iron deficiency anaemia [53]. Of note, several studies have indicated that the association of poor haemoglobin and/or iron status with vitamin D deficiency may be mutual [28,54,55]. The exact mechanisms for the association between anaemia or iron deficiency anaemia and vitamin D status are not known. However, there is evidence that the synthesis of 25(OH)D_3_ from cholecalciferol (vitamin D_3_) in the liver requires hydroxylation which depends on cytochrome P-450 25-hydroxylase (CYP2R1), a heme-containing enzyme, and thus, it is likely that iron deficiency might impair the synthesis of vitamin D_3_ leading to mild vitamin D deficiency [56]. Poor iron status may also impair vitamin D metabolism by increasing the expression of fibroblast growth factor 23 (FGF23), a bone-derived hormone which converts 25(OH)D and 1,25(OH)_2_D into inactive metabolites [57,58]. In our study, however, we did not find any association of iron deficiency with vitamin D insufficiency and/or vitamin D deficiency. The lack of association between iron status and vitamin D insufficiency or deficiency in our study could be due to inadequate samples with iron deficiency.

Our findings also showed that pregnant women with sub-optimal/marginal vitamin A status were 2.30 times more likely to have vitamin D deficiency than pregnant women with adequate vitamin A status. However, marginal vitamin A status was not associated with vitamin D insufficiency in this study population. We are unable to compare the present findings with others as we could not find any study that has reported the association between vitamin A status and vitamin D deficiency. To the best of our knowledge, this is the first study that has identified poor vitamin A status as a risk factor of vitamin D deficiency. One possible explanation for the present finding is that vitamin A plays a key role in signalling the vitamin D pathway. There has been a suggestion that 9-cis-retinoic acid, an active vitamin A metabolite, and the ligand of retinoid X receptor, form a heterodimer complex with vitamin D receptor (VDR), which helps VDR signalling and suppresses the degradation of circulating vitamin D [59,60].

An earlier study in Bangladesh showed a higher prevalence of vitamin D deficiency (defined as 25(OH)D < 37.5 nmol/L) among women from a low socio-economic group compared to women from a high socio-economic group [15]. Among the various socio-economic factors, we found that pregnant women whose husbands were farmers were at higher risk of vitamin D insufficiency than pregnant women whose husbands were in business or service. However, no significant association between a husband’s occupation and vitamin D deficiency was observed. While difficult to explain the present finding, the vitamin D status of a farmer’s wife may be influenced by other factors that are not measured in the present study.

In this study, we did not find any association between the frequency of intake of selected vitamin D-rich foods and vitamin D status. This was similar to the finding of a study among garment factory workers in Bangladesh [17]. Consistent with this, a study carried out with pregnant women, and their husbands living in Dhaka city showed that the availability of vitamin D-rich foods in the home was an unimportant predictor of vitamin D status [20]. It is important to recognise that a diet containing natural vitamin D provides only about 10 per cent of the body’s vitamin D requirements [61] and is considered a poor predictor of vitamin D status [62]. Furthermore, the consumption of vitamin D fortified foods and vitamin D supplements is known to improve vitamin D status [29,61]. In Bangladesh, vitamin D fortified foods are limited [20] and are highly unlikely to be available in rural areas. Besides, none of the pregnant women in our study reported taking vitamin D supplements (data not shown). Of note, vitamin D supplementation is currently not recommended by the Bangladesh national strategy for prevention and control of vitamin D deficiencies [63]. It is also noteworthy that 2016 WHO guidelines for antenatal care did not recommend routine supplementation with vitamin D for pregnant women, but mentioned that “for pregnant women with documented vitamin D deficiency, vitamin D supplements may be given at the current recommended nutrient intake of 200 IU (5 µg) per day” [64]. Therefore, the present findings emphasise the need for active consideration for low dose vitamin D supplementation for pregnant women as a short-term preventive strategy.

The strength of our study is that we have considered the effect of subclinical infection/inflammation for the first time, while assessing the vitamin D status in this population. Besides, we have identified the risk factors of vitamin D deficiency and insufficiency separately after controlling for potential confounders. This study has some limitations that should be considered in interpreting the results. Firstly, we used convenience sampling for selecting the study participants and thus, the findings of this study may represent the wider population from which participants were drawn. Furthermore, since the data were collected in 2015, there may be a slight possibility that the findings of this study may not reflect the present situation. However, it is likely that for most pregnant women in this population, the situation is no better, overall, than that reported here. Secondly, the dietary data were focused on the frequency of consumption, not on the actual amount of consumption. Third, we do not have information regarding time spent outdoors or exposure of skin to sunlight which is an important factor for endogenous production of vitamin D_3_. It is important to note, however, that a large majority of the women in rural Bangladesh cover their body by wearing the burka and/or veil for either cultural or religious reasons.

## 5. Conclusions

Vitamin D deficiency and insufficiency are highly prevalent among pregnant rural women in Bangladesh. The risk of both vitamin D deficiency and insufficiency was significantly higher in nulliparous pregnant women and women in their first trimester of pregnancy. Furthermore, a husband’s occupation and anaemia status appear to be important predictors of vitamin D insufficiency. Nevertheless, younger age and sub-optimal vitamin A status are the potential risk factors of vitamin D deficiency in this population. Our results suggest the need for a comprehensive intervention strategy, including the consideration of low-dose vitamin D supplementation and ways to improve haemoglobin and vitamin A status to prevent vitamin D deficiency in this population. Further studies with larger sample size are recommended to explore the association between anaemia and/or iron deficiency and vitamin D status in the future. 

## Figures and Tables

**Table 1:** Characteristics of the pregnant women who participated in the study.

Variable	*n*	%
**Age (Year)**		
<20	124	24.0
20–24.0	163	31.7
≥25	228	44.3
**Parity**		
Nullipara	191	37.1
Multipara (parity ≥ 1)	324	62.9
**Gestational Age (Week)**		
<13	70	13.6
≥13	445	86.4
**Participant’s Education**		
Functionally illiterate *	229	44.5
Class 6–10 but not SSC **	201	39.0
SSC or above	85	16.5
**Husband’s Education**		
Functionally illiterate *	299	58.1
Class 6–10 but not SSC **	109	21.1
SSC or above	107	20.8
**Husband’s Occupation**		
Labourer	198	38.4
Farmer	136	26.4
Business	108	21.0
Service	73	14.2
**Participant’s Occupation**		
No	496	96.3
Yes	19	3.7
**Household Size**		
Small family (<5)	310	60.2
Large family (≥5)	205	39.8
**Cultivable Land Ownership**		
No land	291	56.5
Small land holding	224	43.5
**Vitamin/Mineral Supplementation**		
Yes	40	7.8
No	475	92.2
**Presence of Inflammation/Sub-Clinical Infection**		
Acute (judged by serum CRP > 10.0 mg/L)	37	7.2
Chronic (judged by serum AGP > 1.0 g/L)	8	1.6
**Selected Micronutrient Status**		
Anaemia (Haemoglobin < 11.0 g/dL)	176	34.2
Iron deficiency (Serum ferritin < 15.0 μg/dL)	136	26.4
Sub-optimal vitamin A status (Serum retinol < 30.0 μg/dL)	155	30.1

* Never been to school or completed up to Class V. ** Secondary School Certificate.

**Table 2:** Differences in mean vitamin D concentrations among pregnant rural women by age, parity and gestational age, inflammation/infection and selected micronutrient status group.

Variable	*n*	Mean	SD	*p*-Value
**Age (year)**				
<20	124	40.1 ^a^	13.7	0.001 *
20–24	163	43.3 ^a^	14.7	
≥25	228	50.2 ^b^	18.1	
**Parity**				
Nullipara	191	39.8	13.7	0.001
Multipara (parity ≥ 1)	324	49.0	17.2	
**Gestational age (week)**				
<13	70	40.4	12.7	0.005
≥13	445	46.4	17.0	
**Taking Vitamin/Mineral supplement**				
Yes	40	48.1	18.4	0.322
No	475	45.4	16.4	
**Acute inflammation/infection**				
Elevated serum CRP (>10.0 mg/L)	37	46.1	18.3	0.848
Normal serum CRP (<10.0 mg/L)	478	45.5	16.5	
**Chronic inflammation/infection**				
Elevated serum AGP (>1.0 g/L)	8	49.6	17.9	0.493
Normal serum AGP (<1.0 g/L)	507	45.5	16.6	
**Anaemia status**				
Anaemic (Haemoglobin < 11.0 g/dL)	176	44.5	15.5	0.282
Normal (Haemoglobin ≥ 11.0 g/dL)	339	46.1	17.1	
**Iron Status**				
Deficient (Serum ferritin < 15.0 μg/dL)	136	44.5	15.1	0.369
Normal (Serum ferritin ≥ 15.0 μg/dL)	379	46.0	17.1	
**Vitamin A Status**				
Sub-optimal (Serum retinol < 30.0 μg/dL)	155	41.9	15.8	0.001
Normal (Serum retinol ≥ 30.0 μg/dL)	360	47.1	16.7	

Groups were compared by independent *t*-test. * One-way ANOVA followed by LSD for sub-group comparison. Different superscripts were significantly different.

**Table 3:** Differences in the prevalence of vitamin D deficiency and insufficiency among pregnant rural women by various socio-demographic, dietary and pregnancy-related factors.

Variable	Normal		Insufficiency		Deficiency		*p*-Value
	*n*	%	*n*	%	*n*	%	
**Age (year)**							
<20	28	15.3	66	27.2	30	33.7	
20–24	48	26.2	82	33.7	33	37.1	0.000
≥25	107	58.5	95	39.1	26	29.2	
**Parity**							
Nullipara	41	22.4	103	42.4	47	52.8	
Multiparous (≥1)	142	77.6	140	57.6	42	47.2	0.000
**Gestational age (week)**							
<13	15	8.2	39	16.0	16	18.0	
≥13	168	91.8	204	84.0	73	82.0	0.026
**Participant’s education**							
Functionally illiterate *	88	48.1	108	44.4	33	37.1	
Class 6–10 but not SSC **	63	34.4	100	41.2	38	42.7	0.319
SSC or above	32	17.5	35	14.4	18	20.2	
**Husband’s education**							
Functionally illiterate *	113	61.7	134	55.1	52	58.5	
Class 6–10 but not SSC **	30	16.4	61	25.1	18	20.2	0.308
SSC or above	40	21.9	48	19.8	19	21.3	
**Husband’s occupation**							
Labourer	74	40.4	91	37.5	33	37.1	
Farmer	38	20.8	77	31.7	21	23.6	
Business	45	24.6	46	18.9	17	19.1	
Service	26	14.2	29	11.9	18	20.2	0.114
**Participant’s occupation**							
Service	6	3.3	9	3.7	4	4.5	
Housewife	177	96.7	234	96.3	85	95.5	0.371 ***
**Household size**							
Small family (<5)	122	66.7	141	58.0	47	52.8	
Large family (≥5)	61	33.3	102	42.0	42	47.2	0.058
**Cultivable land ownership**							
No land	111	60.7	134	55.1	46	51.7	
Small land holding	72	39.3	109	44.9	43	48.3	0.319
**Intake of vitamin D rich food**							
Milk and milk products							
<3	100	54.6	125	51.4	41	46.1	
≥3	83	45.4	118	48.6	48	53.9	0.419
Eggs							
<3	89	48.6	113	46.5	39	43.8	
≥3	94	51.4	130	53.5	50	56.2	0.751
Big fish							
<3	124	67.8	174	71.6	70	78.7	
≥3	59	32.2	69	28.4	19	21.3	0.175
Meat and Liver							
<1	97	53.0	139	57.2	47	52.8	
≥1	86	47.0	104	42.8	42	47.2	0.636
**Vitamin/Mineral supplementation**							
Yes	15	8.2	19	7.8	6	6.7	
No	168	91.2	224	92.2	83	93.3	0.952

* Never been to school or completed up to Class V. ** Secondary School Certificate. *** Exact test.

**Table 4:** Differences in the prevalence of vitamin D deficiency and insufficiency among pregnant rural women by the presence of acute and chronic inflammation/infection and selected micronutrient deficiencies.

Variable	Normal		Insufficiency		Deficiency		*p*-Value
	*n*	%	*n*	%	*n*	%	
**Acute inflammation/infection**							
Elevated serum CRP (>10.0 mg/L)	15	8.2	14	5.8	8	9.0	
Normal serum CRP (<10.0 mg/L)	168	91.8	229	94.2	81	91.0	0.498
**Chronic inflammation/infection**							
Elevated serum AGP (>1.0 g/L)	4	2.2	3	1.2	1	1.1	
Normal serum AGP (<1.0 g/L)	179	97.8	240	98.8	88	98.9	0.805 *
**Anaemia status**							
Anaemic (Haemoglobin < 11.0 g/dL)	56	30.6	90	37.0	30	33.7	
Normal (Haemoglobin ≥ 11.0 g/dL)	127	69.4	153	63.0	59	66.3	0.381
**Iron Status**							
Deficient (Serum ferritin < 15.0 μg/dL)	45	24.6	69	28.4	22	24.7	
Normal (Serum ferritin ≥ 15.0 μg/dL)	138	75.4	174	71.6	67	75.3	0.625
**Vitamin A Status**							
Sub-optimal (Serum retinol < 30.0 μg/dL)	45	24.6	72	29.6	38	42.7	
Normal (Serum retinol ≥ 30.0 μg/dL)	138	75.4	171	70.4	51	57.3	0.009

* Exact test.

**Table 5:** Logistic regression analysis (odds ratios) for vitamin D deficiency and insufficiency among pregnant rural women in Bangladesh.

Variable	Vitamin D Insufficiency *			Vitamin D Deficiency **		
	OR	95% CI	*p*-Value	OR	95% CI	*p*-Value
**Age (year)**						
<25	-	-	-	2.12	1.06–4.21	0.033
≥25 (reference)	-			-		
**Parity**						
Nulliparous	2.72	1.75–4.23	0.0001	2.65	1.34–5.25	0.005
Multipara (reference)	-			-		
**Gestational age (week)**						
<13	2.68	1.39–5.19	0.003	2.55	1.12–5.79	0.025
≥13 (reference)	-			-		
**Husband’s occupation**						
Labourer	1.26	0.80–2.00	0.332	-	-	-
Farmer	2.06	1.22–3.50	0.007			
Business/Service (reference)	-			-		
**Anaemia status**						
Anaemic (Haemoglobin < 11.0 g/dL)	1.53	0.99–2.35	0.056	-	-	-
Normal (Haemoglobin ≥ 11.0 g/dL) (reference)						
**Vitamin A Status**						
Sub-optimal (Serum retinol < 30.0 μg/dL)	-	-	-	2.30	1.28–4.11	0.005
Normal (reference)	-					

* 25(OH)D < 30.0 nmol/L; ** 25(OH)D 30.0–50.0 nmol/L.

## Data Availability

The data presented in this study are available on request from the corresponding author.

## References

[1] Holick M.F. (2002). Vitamin D: The underappreciated D-lightful hormone that is important for skeletal and cellular health. Curr. Opin. Endocrinol. Diabetes Obes..

[2] Autier P., Boniol M., Pizot C., Mullie P. (2014). Vitamin D status and ill health: A systematic review. Lancet Diabetes Endocrinol..

[3] Holick M.F. (2004). Vitamin D: Importance in the prevention of cancers, type 1 diabetes, heart disease, and osteoporosis. Am. J. Clin. Nutr..

[4] Iolascon G., Letizia Mauro G., Fiore P., Cisari C., Benedetti M.G., Panella L., de Sire A., Calafiore D., Moretti A., Gimigliano F. (2018). Can vitamin D deficiency influence muscle performance in post-menopausal women? A multicenter retrospective study. Eur. J. Phys. Rehabil. Med..

[5] Iolascon G., de Sire A., Calafiore D., Moretti A., Gimigliano R., Gimigliano F. (2015). Hypovitaminosis D is associated with a reduction in upper and lower limb muscle strength and physical performance in post-menopausal women: A retrospective study. Aging Clin. Exp. Res..

[6] Gimigliano F., Moretti A., de Sire A., Calafiore D., Iolascon G. (2018). The combination of vitamin D deficiency and overweight affects muscle mass and function in older post-menopausal women. Aging. Clin. Exp. Res..

[7] Palacios C., Gonzalez L. (2014). Is vitamin D deficiency a major global public health problem?. J. Steroid Biochem. Mol. Biol..

[8] Grover S.R., Morley R. (2001). Vitamin D deficiency in veiled or dark-skinned pregnant women. Med. J. Aust..

[9] Sachan A., Gupta R., Das V., Agarwal A., Awasthi P.K., Bhatia V. (2005). High prevalence of vitamin D deficiency among pregnant women and their newborns in northern India. Am. J. Clin. Nutr..

[10] Brannon P.M. (2012). Vitamin D and adverse pregnancy outcomes: Beyond bone health and growth. Proc. Nutr. Soc..

[11] Bener A., AL-Hamaq A.O.A.A., Saleh N.M. (2013). Association between vitamin D insufficiency and adverse pregnancy outcome: Global comparisons. Int. J. Womens Health.

[12] Bodnar L.M., Catov J.M., Zmuda J.M., Cooper M.E., Parrott M.S., Roberts J.M., Marazita M.L., Simhan H.N. (2010). Maternal Serum 25-Hydroxyvitamin D Concentrations Are Associated with Small-for-Gestational Age Births in White Women. J. Nutr..

[13] Roth D.E., Leung M., Mesfin E., Qamar H., Watterworth J., Papp E. (2017). Vitamin D supplementation during pregnancy: Current and future state of the evidence from a systematic review of randomized controlled trials. BMJ.

[14] Islam M.Z., Shamim A.A., Kemi V., Nevanlinna A., Akhtaruzzaman M., Laaksonen M., Jehan A.H., Jahan K., Khan H.U., Lamberg-Allardt C. (2008). Vitamin D deficiency and low bone status in adult female garment factory workers in Bangladesh. Br. J. Nutr..

[15] Islam M.Z., Lamberg-Allardt C., Kärkkäinen M., Outila T., Salamatullah Q., Shamim A.A. (2002). Vitamin D deficiency: A concern in premenopausal Bangladeshi women of two socio-economic groups in rural and urban region. Eur. J. Clin. Nutr..

[16] Islam M.Z., Akhtaruzzaman M., Lamberg-Allardt C. (2006). Hypovitaminosis D is common in both veiled and nonveiled Bangladeshi women. Asia Pac. J. Clin. Nutr..

[17] Mahmood S., Rahman M., Biswas S.K., Saqueeb S.N., Zaman S., Manirujjaman M., Perveen R., Ali N. (2017). Vitamin D and Parathyroid Hormone Status in Female Garment Workers: A Case-Control Study in Bangladesh. BioMed Res. Int..

[18] Ahmed A.K.M.S., Haque W.M.M.U.I., Uddin K.N., Abrar F.A., Afroz F., Huque H.F., Afrose S.R. (2018). Vitamin D and bone mineral density status among postmenopausal Bangladeshi women. IMC J. Med. Sci..

[19] Asaduzzaman M., Basak M.R., Islam M.S., Juliana F.M., Ferdous T., Islam M.J., Al-Mamun A., Sabrina S., Uddin M.M., Islam M.K. (2018). Vitamin D Deficiency and Insufficiency in Healthy Pregnant Women Living in Dhaka, Bangladesh. IOSR J. Dent. Med. Sci..

[20] Jeong J.-H., Korsiak J., Papp E., Shi J., Gernand A.D., Mahmud A.A., Roth D.E. (2019). Determinants of Vitamin D Status of Women of Reproductive Age in Dhaka, Bangladesh: Insights from Husband–Wife Comparisons. Curr. Dev. Nutr..

[21] Bhowmik B., Siddique T., Majumder A., Mdala I., Hossain I.A., Hassan Z., Jahan I., Moreira N.C.V., Alim A., Basit A. (2019). Maternal BMI and nutritional status in early pregnancy and its impact on neonatal outcomes at birth in Bangladesh. BMC Pregnancy Childbirth.

[22] Schulze K.J., Shaikh S., Ali H., Shamim A.A., Wu L.S.-F., Mitra M., Arguello M.A., Kmush B., Sungpuag P., Udomkesmelee E. (2019). Antenatal Multiple Micronutrient Supplementation Compared to Iron–Folic Acid Affects Micronutrient Status but Does Not Eliminate Deficiencies in a Randomized Controlled Trial Among Pregnant Women of Rural Bangladesh. J. Nutr..

[23] Holvik K., Meyer H., Haug E., Brunvand L. (2005). Prevalence and predictors of vitamin D deficiency in five immigrant groups living in Oslo, Norway: The Oslo Immigrant Health Study. Eur. J. Clin. Nutr..

[24] Van der Meer I.M., Karamali N.S., Boeke A.J.P., Lips P., Middelkoop B.J., Verhoeven I., Wuister J.D. (2006). High prevalence of vitamin D deficiency in pregnant non-Western women in The Hague, Netherlands. Am. J. Clin. Nutr..

[25] Dawodu A., Absood G., Patel M., Agarwal M., Ezimokhai M., Abdulrazzaq Y., Khalayli G. (1998). Biosocial factors affecting vitamin D status of women of childbearing age in the United Arab Emirates. J. Biosoc. Sci..

[26] Joh H.-K., Lim C.S., Cho B. (2015). Lifestyle and Dietary Factors Associated with Serum 25-Hydroxyvitamin D Levels in Korean Young Adults. J. Korean Med. Sci..

[27] Jääskeläinen T., Knekt P., Marniemi J., Sares-Jäske L., Männistö S., Heliövaara M., Järvinen R. (2013). Vitamin D status is associated with sociodemographic factors, lifestyle and metabolic health. Eur. J. Nutr..

[28] Azizi-Soleiman F., Vafa M., Abiri B., Safavi M. (2016). Effects of iron on vitamin d metabolism: A systematic review. Int. J. Prev. Med..

[29] Roth D.E., Abrams S.A., Aloia J., Bergeron G., Bourassa M.W., Brown K.H., Calvo M.S., Cashman K.D., Combs G., De-Regil L.M. (2018). Global prevalence and disease burden of vitamin D deficiency: A roadmap for action in low- and middle-income countries. Ann. N. Y. Acad. Sci..

[30] Haynes B.M.H., Pfeiffer C.M., Sternberg M.R., Schleicher R.L. (2013). Selected Physiologic Variables Are Weakly to Moderately Associated with 29 Biomarkers of Diet and Nutrition, NHANES 2003–2006. J. Nutr..

[31] Duncan A., Talwar D., McMillan D.C., Stefanowicz F., O’Reilly D.S.J. (2012). Quantitative data on the magnitude of the systemic inflammatory response and its effect on micronutrient status based on plasma measurements. Am. J. Clin. Nutr..

[32] Ahmed F., Hasan N., Kabir Y. (1997). Vitamin A deficiency among adolescent garment factory workers in Bangladesh. Eur. J. Clin. Nutr..

[33] Bouillon R. (2017). Comparative analysis of nutritional guidelines for vitamin D. Nat. Rev. Endocrinol..

[34] Giustina A., Adler R.A., Binkley N., Bouillon R., Lazaretti-Castro E.M., Marcocci C., Rizzoli R., Sempos C.T., Bilezikian J.P. (2019). Controversies in Vitamin D: Summary Statement from an International Conference. J. Clin. Endocrinol. Metab..

[35] United Nations Children’s Fund, United Nations University, World health Organization (2001). Iron Deficiency Anaemia Assessment, Prevention, and Control: A Guide for Programme Managers.

[36] Tomkins A. (2003). Assessing micronutrient status in the presence of inflammation. J. Nutr..

[37] Thurnham D.I., McCabe L.D., Haldar S., Wieringa F.T., Northrop-Clewes C.A., McCabe G.P. (2010). Adjusting plasma ferritin concentrations to remove the effects of subclinical inflammation in the assessment of iron deficiency: A meta-analysis. Am. J. Clin. Nutr..

[38] Willumsen J.F., Simmank K., Filteau S.M., Wagstaff L.A., Tomkins A.M. (1997). Toxic damage to the respiratory epithelium induces acute phase changes in vitamin A metabolism without depleting retinol stores of South African children. J. Nutr..

[39] Thurnham D.I., McCabe G.P., Northrop-Clewes C.A., Nestel P. (2003). Effects of subclinical infection on plasma retinol concentrations and assessment of prevalence of vitamin A deficiency: Meta-analysis. Lancet.

[40] World Health Organization (2009). Global prevalence of vitamin A deficiency in populations at risk 1995–2005. WHO Global Database on Vitamin A Deficiency.

[41] Yoo E.-H., Cho H.-J. (2015). Prevalence of 25-hydroxyviotamin D deficiency in Korean patients with anemia. J. Clin. Lab. Anal..

[42] Sahu M., Bhatia V., Aggarwal A., Rawat V., Saxena P., Pandey A., Das V. (2009). Vitamin D deficiency in rural girls and pregnant women despite abundant sunshine in northern India. Clin. Endocrinol..

[43] Wang J., Yang F., Mao M., Liu D.-H., Yang H.-M., Yang S.-F. (2010). High prevalence of vitamin D and calcium deficiency among pregnant women and their newborns in Chengdu, China. World J. Pediatr..

[44] Johnson D.D., Wagner C.L., Hulsey T.C., McNeil R.B., Ebeling M., Hollis B.W. (2011). Vitamin D deficiency and insufficiency is common during pregnancy. Am. J. Perinatol..

[45] Parlak M., Kalay S., Kalay Z., Kirecci A., Guney O., Koklu E. (2015). Severe vitamin D deficiency among pregnant women and their newborns in Turkey. J. Matern. Fetal Neonatal Med..

[46] Holmes V.A., Barnes M.S., Alexander H.D., McFaul P., Wallace J.M. (2009). Vitamin D deficiency and insufficiency in pregnant women: A longitudinal study. Br. J. Nutr..

[47] Lykkedegn S., Beck-Nielsen S.S., Sorensen G.L., Andersen L.B., Fruekilde P.B.N., Nielsen J., Kyhl H.B., Joergensen J.S., Husby S., Christesen H.T. (2017). Vitamin D supplementation, cord 25-hydroxyvitamin D and birth weight: Findings from the Odense Child Cohort. Clin. Nutr..

[48] Vandevijvere S., Amsalkhir S., Van Oyen H., Moreno-Reyes R. (2012). High prevalence of vitamin D deficiency in pregnant women: A national cross-sectional survey. PLoS ONE.

[49] Cross N.A., Hillman L.S., Allen S.H., Krause G.F., Vieira N.E. (1995). Calcium homeostasis and bone metabolism during pregnancy, lactation, and postweaning: A longitudinal study. Am. J. Clin. Nutr..

[50] Moon R.J., Davies J.H., Cooper C., Harvey N.C. (2020). Vitamin D, and Maternal and Child Health. Calcif. Tissue Int..

[51] Mulligan M.L., Felton S.K., Riek A.E., Bernal-Mizrachi C. (2010). Implications of vitamin D deficiency in pregnancy and lactation. Am. J. Obstet. Gynecol..

[52] Bowyer L., Catling-Paull C., Diamond T., Homer C., Davis G., Craig M.E. (2009). Vitamin D, PTH and calcium levels in pregnant women and their neonates. Clin. Endocrinol..

[53] Jin H.J., Lee J.H., Kim M.K. (2013). The prevalence of vitamnin D deficiency in iron-deficient and normal children under the age of 24 months. Blood Res..

[54] Malczewska-Lenczowska J., Sitkowski D., Surała O., Orysiak J., Szczepánska B., Witek K. (2018). The Association between Iron and Vitamin D Status in Female Elite Athletes. Nutrients.

[55] Thomas C.E., Guillet R., Queenan R.A., Cooper E.M., Kent T.R., Pressman E.K., Vermeylen F.M., Roberson M.S., O’Brien K.O. (2015). Vitamin D status is inversely associated with anemia and serum erythropoietin during pregnancy. Am. J. Clin. Nutr..

[56] Toxqui L., Vaquero M.P. (2015). Chronic Iron Deficiency as an Emerging Risk Factor for Osteoporosis: A Hypothesis. Nutrients.

[57] Bergwitz C., Jüppner H. (2010). Regulation of Phosphate Homeostasis by PTH, Vitamin D, and FGF23. Annu. Rev. Med..

[58] Clinkenbeard E.L., Farrow E.G., Summers L.J., Cass T.A., Roberts J.L., Bayt C.A., Lahm T., Albrecht M., Allen M.R., Peacock M. (2014). Neonatal iron deficiency causes abnormal phosphate metabolism by elevating FGF23 in normal and ADHR mice. J. Bone Miner. Res..

[59] Sanchez-Martinez R., Castillo A.I., Steinmeyer A., Aranda A. (2006). The retinoid X receptor ligand restores defective signalling by the vitamin D receptor. EMBO Rep..

[60] Khosravi-Boroujeni H., Ahmed F., Sarrafzadegan N. (2016). Is association between vitamin D and Metabolic syndrome independent of other micronutrients?. Int. J. Vitam. Nutr. Res..

[61] Dawodu A., Wagner C.L. (2007). Mother-child vitamin D deficiency: An international perspective. Arch. Dis. Child..

[62] Hollick M.F., Chen T.C. (2008). Vitamin D deficiency: A worldwide problem with health consequences. Am. J. Clin. Nutr..

[63] Institute of Public Health Nutrition (2015). National Strategy on Prevention and Control of Micronutrient Deficiencies, Bangladesh (2015–2024).

[64] WHO (2016). Recommendations on Antenatal Care for a Positive Pregnancy Experience.

